# An Enquiry into the Morbid Effects of Deficiency of Food, Chiefly with Reference to Their Occurrence among the Destitute Poor; Also Practical Observations on the Treatment of Such Cases

**Published:** 1839-10

**Authors:** 


### Art. XVI.

*An Inquiry into the Morbid Effects of Deficiency of Food, chiefly with reference to their occurrence among the Destitute Poor; also Practical Observations on the Treatment of such cases.* By Richard Baron Holland, m.d.—*London*, 1839. 8vo, pp. 77.

When we learn, from some good people, that man may live well on a vegetable diet, and that his clay requires nought but water to moisten it, instead of gainsaying them, we refer them to the corn mart, in Mark Lane, or to some other assemblage of tillers of the soil, for specimens of the connexion of air and exercise, meat and beer, with large, athletic, vigorous, healthy forms. And when we find some of our well-meaning acquaintance educating their children in habits of abstinence and water, and complaining that, notwithstanding their careful dietary, the little growths are not so vegetative as they could wish, we have often found reason to congratulate them, that the daily allowance of a glass of stout and a little more meat has entirely altered the appearance of things; has put an end to a catalogue of pains, and aches, and disorders; has coloured the pale cheek, and has made what promised to become a rickety bantling into a reasonably healthy boy. Why mankind requires the diet which we should recommend is a question which would be better answered by an experienced breeder of cattle than by any one beside. At present we are disposed to be dogmatic; and to say that, in the great majority of cases, whether from the habit of parents having communicated a particular disposition to their offspring, or from the effects of injudicious general intellectual and physical management, the strongest organization will not be raised in this country on oatmeal and potatoes—add to these as much water as you may—but on good meat and bread, with *genuine* infusion of malt and hops: we say "genuine," because the scandal which attaches itself to beer is—sometimes at least—owing to the poisoned compound which it too often pleases and profits brewers to sell under that name.

Dr. Holland has written a useful book, and one which merits, and, we trust, will receive a more extended application than he has chosen to make of it. Connected with the Royal Infirmary and Poorhouse of Manchester for more than eight years, he has thereby enjoyed considerable opportunities of studying the subject of his little work. The habits of the manufacturing poor of Manchester are those of manufacturers generally. They are improvident, careless in their diet, clothing, and lodging; confined almost wholly in a vitiated atmosphere; wearied with long-continued exertion, and never refreshed by sufficient repose; intemperate in the use of fermented liquors, for the sake of which they deny themselves wholesome food, and live on that which is bad and innutritious. The consequences of such a mode of life are, that "the digestive organs become impaired, and the function of digestion is so feebly and imperfectly performed, that even much less nutritive matter is extracted from the indigestible and impoverished diet they use than would be the case if the stomach and its appendages were in a healthy and vigorous condition—a disordered state of the mucous membrane of the alimentary canal is produced; it becomes irritable and morbidly sensitive—the secretions are vitiated, profuse, or defective, and abdominal pains, diarrhœa, or constipation is the consequence." (p. 6.)

The symptoms produced by deficiency of food are carefully noticed by Dr. Holland. In cases of very gradual starvation, an urgent feeling of hunger is not a prominent symptom; and even when it exists at first, it usually soon diminishes, and is succeeded by a feeling of exhaustion and faintness, and even by loathing of food, if the abstinence has been long protracted. The mental condition connected with poverty is mentioned as in part accounting for this deficiency of appetite. A morbid state of the nervous system is among the most interesting circumstances connected with deficiency of food. We do not know any subject of more practical importance than this in all its bearings, and we shall quote Dr. Holland's remarks upon it.

"The depression produced on the nervous system is very early manifested in the impaired energies of all the vital functions, the weakened conditions of the intellectual faculties and moral feelings, and diminution of the general sensibility. Disturbance of the cerebral functions is at first shown by an unnatural languor, despondency, and listlessness; slowness and hebetude of intellect, with an inability to employ the thoughts steadily and profitably on any subject. Notwithstanding all this general languor, however, the patient sometimes manifests a highly nervous state; he is startled by any sudden noise, and hurried by the most trifling occurrences. He is liable to attacks of giddiness, 'swimming in the head,' staggering, dimness of sight, with temporary delirium, and either falls as in an apoplectic fit, or lapses gradually from a lethargic state into one of stupor, or even of complete coma. In many respects, the symptoms in these cases have considerable resemblance to the effects of exposure to cold. Mania, or mental imbecility, has sometimes been produced by defective nutrition..... In consequence of the torpor of the brain and intellectual faculties, it is often extremely difficult to obtain the requisite information from patients. Instead of showing any anxiety to communicate the symptoms and cause of their illness, or to relate the privations they have undergone, they generally have an unwillingness to be questioned—lie in a listless or lethargic state, without taking any notice of what is going on, and seem desirous only not to be disturbed. .... Careful observation has convinced me that the listlessness and torpor of the mental faculties—the tendency to fainting or to perfect syncope, and, finally, a state of cerebral oppression, amounting in some cases to coma, are among the most characteristic symptoms of defective nutrition, and the surest indication of its existence to a serious extent. The effects too on the moral feelings are very striking, and often truly deplorable." (pp. 26-30.)

Dr. Holland observes that the symptoms which he has mentioned as characterizing a deficient nutrition vary from those which are commonly associated with that state; but it must be borne in mind that other causes, of a depressing character, to which we have already alluded, in addition to deficiency of food, are in constant operation on those individuals from whom his observations are taken. But there is a multitude of cases of minor degrees of suffering, in which the symptoms are less marked than above mentioned.

"Such a state is indicated by a sallow and dingy appearance of the skin, a soft and flabby feeling of the flesh, more or less emaciation, general debility, feebleness of the circulation, and frequently swelling of the ankles. The stomach becomes disordered, the appetite defective, and digestion impaired. The individual feels languid and desponding, is soon fatigued, incapable of exertion, and has an irresistible disposition to fall asleep, from which he is apt to awake suddenly and in a fright. The body is easily chilled, breathlessness and palpitation are experienced after slight exertion; attacks of vertigo, tinnitus aurium, and transient blindness, are common, and there is a peculiar forlorn and dejected aspect of countenance which is very characteristic. This state of things is commonly soon succeeded by some specific disease, though it sometimes continues, with only slight variation, for a very protracted period, until the patient falls by slow degrees into a state of mental as well as physical incapacity, and, being no longer able to procure any employment, is completely invalided, and applies for medical relief." (pp. 33-4.)

The above remarks must be regarded as applying to the less frequent cases of the effects of deficient nutriment. The instances in which this deficiency acts as a predisposing cause of many diseases are far more numerous. In a political view, the condition of the intellectual faculties and moral feelings which is induced by defective nutriment is a subject of the highest importance. And in carrying out any system of education which shall afford a prospect of really bettering the condition of the poorer classes, we entirely agree with Dr. Holland, admitting his saving clause, "that the first step towards improving the mind is to preserve the health, by providing efficiently for the wants of the body." It is now well and, we trust, generally known that a plethoric and an opposite condition of the system may give rise to symptoms nearly resembling each other; the treatment required, however, being different in each. The discrimination of these differences is of great importance, both in states of simple exhaustion and where with this is combined any actual disease. On this subject we shall quote some of Dr. Holland's observations.

"The accession of coma is one of the most severe and fatal signs of exhaustion from defective nutrition. This symptom has been too indiscriminately regarded as indicating a determination of blood to the head or pressure on the brain, and as requiring abstraction of blood for its removal. It must never be forgotten, however, that it arises from a very opposite state of the system—defective circulation, exhaustion or deficiency of nervous power. Coma itself affords no evidence of the state of the brain, unless taken in conjunction with other symptoms and the history of the case. .... I may also mention here a circumstance, which I have often observed, viz., that when coma supervenes towards the termination of diseases of exhaustion, and the pulse becomes slower, it often acquires a degree of fulness and gives an idea of strength, quite at variance with its previous character, and little to have been anticipated from the debilitated state of the system." (pp. 65-9.)

With respect to the treatment of the morbid effects of deficiency of food, it is well known that, in individuals long accustomed to an impoverished diet, no sudden change should be made to food of an opposite character. Such food should be given as will support returning strength, without either burthening the powers of digestion or producing a too stimulant effect. Where exhaustion is so great as to endanger life, much more is required; the horizontal posture should be strictly maintained; heat applied to the surface; diffusible stimulants administered internally.

"If positive coma exist, the sesquicarbonate of ammonia ought to be given with persevering assiduity. We must not be deterred from resolutely using this remedy, with brandy or wine, from any fear that the brain is 'labouring' or congested, or be tempted, with the view of relieving this, to abstract blood at this stage of our treatment; because, whether such be the case or not, that is not the treatment calculated to remove it." (p. 73.)

Symptoms of reaction follow those which indicate the languor produced by exhaustion; such are "flushing of the face, intolerance of light, headach, restlessness, delirium, a dry tongue, and quick pulse." And these require patience and care, frequently with the use of a sedative for their relief.

"During convalescence, from the effects of deficiency of food, congestions are very apt to occur, which require blisters, and sometimes leeches or cupping. This is a natural consequence of the weakened state of the circulation. Pains of a neuralgic character, particularly of the head, are often experienced, for the removal of which, quinine and iron, alone, or in combination with morphia, is generally effectual. If palpitations and irregular action of the heart should be troublesome, they will be most speedily relieved by the judicious combination of tonics and sedatives." (p. 76.)

The subject of treatment of the effects produced by defective nutrition—and under this head we include both food and drink—might have received, with advantage, a more extended notice than Dr. Holland has given to it. There are several points on which, seeing the attention which the author has given to the subject of his essay, we should have been gratified to have had his opinion. According to our experience, there are several classes of individuals, not in good health, to whom Dr. Holland's remarks and observations are especially applicable. To some of these we shall briefly allude. We would, however, premise that, in reasoning on the subject of diet, it should always be borne in mind that men are rarely living in a wholesome state, but are habituated to practices very foreign to health; that the exertions which, in a simple state of society, would have sufficed for maintenance, are not regarded as sufficient now, and that they are too often accompanied by an anxiety and disappointment which are as exhausting as the exertions themselves; and, moreover, that the effects of these and many other circumstances, which must occur to every reflecting practitioner of medicine, are such as diminish and exhaust power. It would be very easy, and on that account it is unnecessary, to give any illustrations of the above remarks. In applying Dr. Holland's observations, we would allude, first, to the poor, who cannot get food convenient for them; secondly, to those who, having been used to a full and somewhat stimulant diet, have, either from necessity or from some conviction of its moral propriety, diminished their allowance to what would be sufficient for those who have never been accustomed to anything better, but which is insufficient for them; and lastly, to those who have always been used to such a diet as would suit the majority, but which is not proportionate to the demands of their constitution, and the exertions, either mental or bodily, to which their daily avocations call them.

A careful investigation of the history of a case, and great attention to both hereditary and acquired constitutional peculiarities, and to temperament, are necessary to the practical application of any dietetic rules to these several classes. But we believe that it is the diet, far more than any medical treatment, that is important in these cases; and, not until diet is made a subject of more permanent importance than is at present the case, will the physician obtain, in the management of the vast class of cases termed nervous, the credit which he assuredly does not at present possess. It has fallen to our lot to observe cases of this kind, of years' duration, and some of the most distressing character, which have been subject to all the systems of simple medical treatment which ingenuity could devise for their relief, but which have speedily, we might almost say instantaneously, been relieved by the discontinuance of medicine, and the employment of diet and regimen instead. We could derive, from facts of this sort, the strongest arguments in favour of change in the present mode of remunerating medical practitioners in this country; such a change, in fact, as should tempt the practitioner, on seeing his patients, to ask himself—instead of "What medicine shall I give in this case?"—"Is it necessary to give any medicine at all?" But we must terminate our notice of Dr. Holland's work by a few remarks illustrative of those which it has already called forth.

The first class of cases which we have mentioned is that treated of by Dr. Holland; and to his judicious observations we have nothing to add. Concerning the second class, we should say, admitting that, with all the conceit and pharisaism which it engenders, the system of total abstinence from fermented liquors is not, in some instances, without its advantages; there are very many persons who have so habituated themselves to a certain stimulus, that it is to the complete sacrifice of their comfort and of the bodily energy which is requisite for the due performance of their duties, for them to discontinue it. We should quite as soon think of recommending such persons to throw off the flannel waistcoat which has saved them from or cured them of rheumatic pains; or the great-coat which has preserved them from shivering throughout the whole winter; or to discontinue the meat breakfast, which has built them up for the entire day, as to abstain from a good allowance of ale or wine; the latter of which, we are assured, on the best authority, is good for the stomach sake and our often infirmities. There are many individuals belonging to the third class; and in these it is frequent to observe some one or more of the symptoms alluded to by Dr. Holland, and which we have quoted. They eat their daily meals with appetite, and the quantity and quality of them are such as would suffice for many; but they suffer from palpitations and intermittent action of the heart, or from very frequent despondency, and sometimes of the most distressing character, or from repeated headachs, increased by the upright posture, or any bodily exertion, or from some other functional derangement. A moderate daily allowance of good beer or wine, with an increase of animal food is a rapid, certain, and perfect cure for these complaints; and a life previously miserable may be thereby rendered joyous and happy.

But we have extended these observations further than was at first our intention, with, however, no regret, as we are perfectly convinced of their practical importance. We conclude by a cordial recommendation of Dr. Holland's valuable little work.

